# Activated Carbon Fiber Cloth/Biomimetic Apatite: A Dual Drug Delivery System

**DOI:** 10.3390/ijms222212247

**Published:** 2021-11-12

**Authors:** Florian Olivier, Sylvie Bonnamy, Nathalie Rochet, Christophe Drouet

**Affiliations:** 1 ICMN, CNRS, Université d’Orléans, UMR 7374, F-45071 Orléans, France; florian.olivier@cnrs-orleans.fr; 2 iBV, CNRS, INSERM, Université Côte d’Azur, F-06107 Nice, France; nathalie.rochet@univ-cotedazur.fr; 3 CIRIMAT, Université Toulouse, CNRS, F-31030 Toulouse, France; christophe.drouet@toulouse-inp.fr

**Keywords:** activated carbon fiber cloth, biomimetic apatite, adsorption, release, tetracycline, aspirin, human osteoblast, bone defect model

## Abstract

A biomaterial that is both bioactive and capable of controlled drug release is highly attractive for bone regeneration. In previous works, we demonstrated the possibility of combining activated carbon fiber cloth (ACC) and biomimetic apatite (such as calcium-deficient hydroxyapatite (CDA)) to develop an efficient material for bone regeneration. The aim to use the adsorption properties of an activated carbon/biomimetic apatite composite to synthetize a biomaterial to be used as a controlled drug release system after implantation. The adsorption and desorption of tetracycline and aspirin were first investigated in the ACC and CDA components and then on ACC/CDA composite. The results showed that drug adsorption and release are dependent on the adsorbent material and the drug polarity/hydrophilicity, leading to two distinct modes of drug adsorption and release. Consequently, a double adsorption approach was successfully performed, leading to a multifunctional and innovative ACC-aspirin/CDA-tetracycline implantable biomaterial. In a second step, in vitro tests emphasized a better affinity of the drug (tetracycline or aspirin)-loaded ACC/CDA materials towards human primary osteoblast viability and proliferation. Then, in vivo experiments on a large cortical bone defect in rats was carried out to test biocompatibility and bone regeneration ability. Data clearly highlighted a significant acceleration of bone reconstruction in the presence of the ACC/CDA patch. The ability of the aspirin-loaded ACC/CDA material to release the drug in situ for improving bone healing was also underlined, as a proof of concept. This work highlights the possibility of bone patches with controlled (multi)drug release features being used for bone tissue repair.

## 1. Introduction

Calcium phosphates (CaPs) have been used in dental and bone surgery since the 1980s, mainly in the form of hydroxyapatite, which has become an essential bone substitute in orthopedic, maxillofacial and implant surgery [1,2,3,4,5]. CaPs are widely used as bone substitutes in various forms, such as granules, dense or porous ceramic pieces, cements and coatings [6,7]. In recent years, biomimetic nanocrystalline apatites have attracted attention due to their chemical composition and (micro)structure close to the mineral phase of bone [8]. Moreover, the occurrence of a hydrated ionic layer on their surface of bone(-like) apatite nanocrystals allows rapid ion exchanges with body fluids [9,10] and a significantly increased reactivity for the adsorption of active (bio)molecules and drugs [11,12]. This is why the synthesis of nanocrystalline biomimetic apatites has been a focus of research, particularly as bone cements [13,14] and coatings [15,16], but also in other domains such as medical imaging [17,18,19] and nanomedicine [20]. Combining drugs with biomaterials is not new in bone engineering [21,22], but the use of biomimetic nanocrystalline apatites as adsorbents is a more recent development [11]. Synthetic nanocrystalline and non-stoichiometric apatites, referred to as “biomimetic”, seem excellent candidates for biomaterial support for in situ delivery systems. These biomimetic apatites have a high surface reactivity, directly related to the presence of the above-mentioned non-apatitic hydrated layer containing labile ions on the nanocrystal surface [11,15,23]. This reactive surface enables the doping of biomimetic apatites with a wealth of active molecules or ions of biological interest. In the literature, various papers have emphasized the role of apatites as a drug delivery system and their use as a carrier of antibiotics [11], antibacterial enzymes [24], bisphosphonate [25,26], growth factor [15,27,28], anticancer drugs [29,30] and bioactive ions [10,31,32,33,34], among others. The possibility of providing additional and new functionalities is a means of adapting the biomaterials used for bone regeneration to support clinical needs and patient well-being [11,15], e.g., to accelerate bone regeneration and limit complications such as infections or bone necrosis.

In contrast, activated carbon fiber cloth (ACC) coated with CaP has not been used, until now, as an in situ delivery system. Applications of ACC were instead mainly developed in the fields of energy as capacitor electrode materials [35,36,37] or in environmental sciences as carbon adsorbents for water treatment [38,39,40], due to their very high specific surface area and related adsorption capacity. However, it is worth noting that activated carbons (also known as charcoal) have long been used as adsorbent materials for water treatment and gas adsorption [41]. They are also used as medical devices for treating overdoses or acute toxicity through oral ingestion [42,43,44].

In this study, ACC is proposed to be used first as a substrate for CaP deposition and then as a novel drug delivery system because of its excellent adsorption properties.

In our previous works, an efficient process for the synthesis of a biomimetic apatite coating on ACC using a sono-electrodeposition process was described [45,46,47]. Using such a process, a biomimetic carbonated calcium-deficient hydroxyapatite (CDA) coating was efficiently deposited on an ACC. It was shown that such ACC/CDA composite biomaterial was biocompatible after being tested in vitro using primary human osteoblasts [47]. The aim of the present work is to take advantage of the adsorption properties of ACC and the reactivity/bioactivity of biomimetic CDA to study the adsorption of different drugs in each component of the ACC/CDA. Two drugs were selected as model drugs, namely tetracycline (TC) and aspirin (acetylsalicylic acid, AA). Tetracycline is a wide-spectrum antibiotic drug that is active against various Gram-negative and Gram-positive bacteria [48,49] and, as such, is a strong antibacterial agent for potentially preventing or treating bone infections. Furthermore, it plays a role in bone regeneration, leading to an increase of collagen type I synthesis, which prevents bone loss and increases bone formation [50]. Aspirin is a non-steroid anti-inflammatory drug (NSAID) that plays an important role in various biological pathways; by modulating the inflammatory response, it may modulate the balance between bone resorption and bone formation [51,52,53,54,55,56]. In this work, in a first step, the adsorption capabilities and release kinetics of tetracycline and aspirin were studied on biomimetic apatite powder and on activated carbon fiber cloth, as well as on a composite ACC/CDA biomaterial. In a second step, the biocompatibility of ACC/CDA-drug(s) materials was evaluated on human osteoblasts. In a third step, these ACC/CDA materials, used as drug delivery systems, were tested on a bone defect model in rats.

## 2. Results and Discussion

### 2.1. Apatite Material Characterization

Two apatite materials were studied: a carbonated calcium-deficient hydroxyapatite (CDA) deposited on ACC substrates and a “reference” nanocrystalline apatite in powder form, which was selected owing to its chemical composition close to that of bone mineral, although non-carbonated. Their chemical, morphological and structural characteristics are reported in Table 1.

SEM observations showed similar features for the two types of apatite, including in particular the usual plate-like morphology for bone-like apatites. Their Ca/P atomic ratio was determined via SEM-Energy dispersive X-ray spectroscopy (EDS). The Ca/P value of 1.46 for the “reference” nanocrystalline apatite was significantly lower than the Ca/P ratio of hydroxyapatite (1.67), therefore stressing the nonstoichiometric character of this apatite compound for bone mineral. The Ca/P value of 1.4 for the carbonated CDA sample was also found to be appreciably lower than 1.67. It should be noted that, in this case, carbonate ions also partially substituted for phosphates ions, thus modifying the P stoichiometry. The Ca/ (P + C) may be estimated to a mean of ~1.35 (noting that the carbonation level (C) varies somewhat over the carbonated CDA sample, as indicated in Table 1), and this value again shows the nonstoichiometry of this biomimetic apatite.

The crystallographic structure determined by X-ray diffraction (Figure S1) shows the occurrence of well-defined Bragg reflections, attributed to the hexagonal hydroxyapatite structure (JCPDS No. 74-0566), with P6_3_/m space group symmetry as described in previous works [47,57]. For the carbonated CDA and the “reference” nanocrystalline apatite, the structural refinement performed allows for the determination of their lattice parameters (*a* and *c*), which are in agreement with the data from the literature [58]. These lattice parameters are characteristic of a nonstoichiometric apatite [57].

### 2.2. Characterization of Activated Carbon Fiber Cloth (ACC) Substrates

First, three ACC substrates (ACC 1, ACC 2 and ACC 3), offering different characteristics in terms of chemical functionalities and porosity, were selected. Their morphological, physico-chemical and textural characteristics were analyzed and are reported in Table 2 Then, they were biologically tested in order to investigate their biocompatibility.

**Figure 1:**
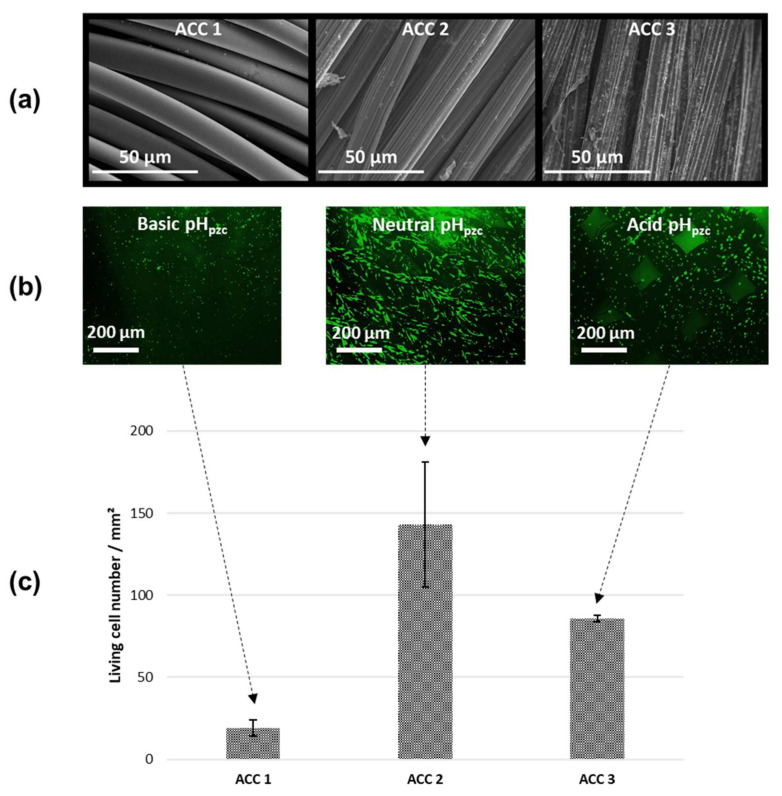
In vitro assessment after 2 days of culture of HOST cells on the three ACC materials. (**a**) SEM images of ACC 1, ACC 2 and ACC 3 substrates; (**b**) fluorescence microscopy images of living cells (green fluorescence) on basic, neutral and acid ACC surfaces; (**c**) living cell number per mm^2^ on ACC surfaces obtained from the fluorescence micrographs.

As reported in Table 2, ACC materials are made of carbon fibers derived from two types of precursors: phenolic resin or viscose. The carbon fiber diameters determined by SEM (Figure 1a) are around 10 µm in ACC 1, range from 8 µm to 12 µm in ACC 2 and from 12 µm to 15 µm in ACC 3. Note that during the industrial process prior to commercialization, carbon fibers may or may not undergo a surface sizing treatment (coating) for facilitating the fiber handling and waving, which we previously identified by X-ray diffraction as zinc aluminate [47]. As for the architecture of the carbon fiber cloths, it consists of twisted carbon fiber threads that are either woven (ACC 1 material) or knitted (ACC 2 and ACC 3 materials), depending on the carbon fiber precursor, to form a flexible material, which is an important characteristic for the medical application of such biomaterials (bone patch).

In view of the drug adsorption study performed in this work, the ACC textural characteristics were investigated. Their specific surface area (S_BET_), calculated from the N_2_ isotherm using the BET (Brunauer, Emmett and Teller) method, and the total pore volume (V_TOTAL_), estimated from the N_2_ amount adsorbed at P/P° = 0.95, were analyzed. ACC 1 and ACC 3 materials displayed a high surface area (ranging from 1440 to 1700 m^2^/g) as compared to ACC 2 (898 m^2^/g). The total pore volume ranged from 0.51 cm^3^/g (ACC 2) to 0.68 cm^3^/g (ACC 1) to 1.27 cm^3^/g (ACC 3), without any direct relationship with the surface area.

The surface chemical functionalities and the pH_pzc_ (pH value corresponding to an ACC net charge of zero) determine the surface hydrophilicity or hydrophobicity of these materials. The pH_pzc_ values varied from acidic, i.e., 5.8 for ACC 3, to almost neutral, i.e., 7.8 for ACC 2, to basic, i.e., 9.4 for ACC 1. The surface chemical functionalities were measured by potentiometric titration, which allows quantification of the amount of acidic functional groups existing at the surface of the ACC materials. As reported in Table 2, their amount varied from 2.93 mmol/g (ACC 2) to 1.31 mmol/g (ACC 3) to 0.48 mmol/g (ACC 1).

The data reported in Table 2 express a large variability of the physico-chemical and textural characteristics of ACCs, which also depicts the potential modularity of the envisioned applications. In this view, it was important to investigate the cell activity and biocompatibility of these three ACC materials before performing drug adsorption experiments.

The cell viability on the three ACC materials was evaluated using primary human osteoblasts (HOST) cultured for two days. Fluorescence images show that living cells (green fluorescence) exhibit a fusiform shape along the carbon fibers only for ACC 2 (Figure 1b). This material has a neutral pH_pzc_ and a high number of acidic functional groups, leading to a hydrophilic surface. For materials displaying a basic (ACC 1) and acidic (ACC 3) pH_pzc_ value and a much lower number of acidic functional groups, osteoblast cells exhibited, in contrast, a round shape on the ACC surface. No dead cells (red fluorescence) were observed at the surface of any of the three materials. After 2 days of culture, the numeration of living cells (Figure 1c) revealed that cell density was higher on ACC 2 when compared to ACC 1 and ACC 3 materials. We hypothesized that the surface chemistry plays an important role in osteoblast viability, and a neutral pH_pzc_ associated with a hydrophilic surface appears to allow the best osteoblast cell affinity.

Therefore, for the drug adsorption experiments described in this paper, the selected ACC material was ACC 2 (FM50K), whose pH_pzc_ of 7.8 is close to the pH of drug solutions (pH = 7.4) and therefore to the physiological pH that is appropriate for the in vivo experiments.

### 2.3. Characterization of ACC/CDA Porosity

**Figure 2:**
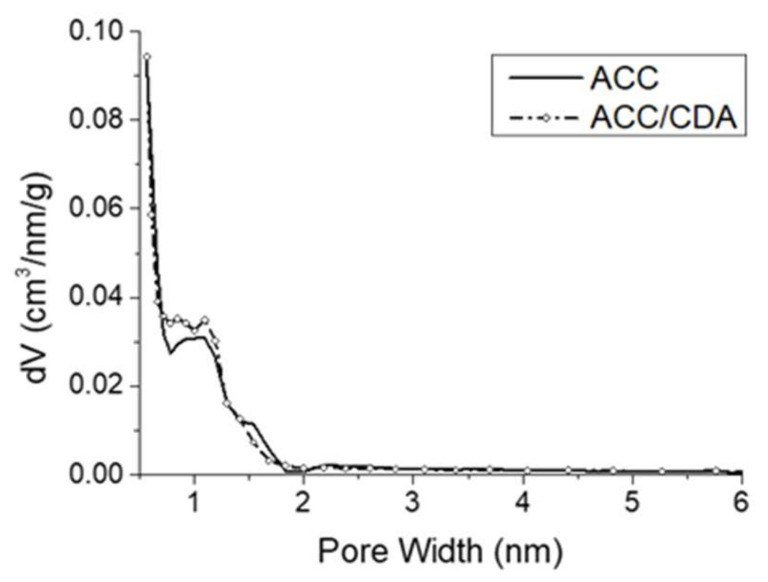
Pore size distribution of ACC and ACC/CDA materials (N_2_ adsorption at 77 K).

Before starting the drug adsorption experiments, it was important to compare the ACC characteristics with and without the CDA coating. Their porous characteristics were studied by N_2_ adsorption at 77 K. The description of the porous volumes and specific surface areas are reported in Table 3, and the pore size distributions are given in Figure 2.

They exhibited similar surface area values; however, a slight increase was observed with the CDA coating: 980 cm^2^/g (ACC/CDA) instead of 898 cm^2^/g (ACC without any coating). This effect can reasonably be attributed to the presence of the nanocrystalline apatite generating additional porosity. These materials are mostly microporous, as seen in their microporous volume, which represents 85% of the total pore volume (ACC material) and 82% of the total pore volume (ACC/CDA material). The two materials presented a similar and narrow pore size distribution, centered around 1 nm, with micropore size ranging from 0.7 nm to 2 nm. A low number of mesopores (2–50 nm in size) was observed, representing less than 18% of the total pore volume. It can be concluded that the porosity is essentially preserved at the nanometric scale when the ACC substrate is coated with hydroxyapatite. These data provide background for the drug adsorption study that follows.

### 2.4. Tetracycline and Aspirin Adsorption Experiments

#### 2.4.1. Adsorption Kinetics and Isotherms on Biomimetic Apatite Powder

The curves of the drug adsorption kinetics report the adsorption capacity (Q_ads_ in µmol of drug/g of apatite) versus contact time (min). The adsorption capacity was calculated using Equation (1):

(1) $$Q_{ads}=\frac{\left(C_{0}-C_{t}\right)\times V}{m}$$

where Q_ads_ is the adsorption capacity at contact time t, C_o_ is the initial drug concentration (µmol/L), C_t_ is the drug concentration at the t contact time (µmol/L), V is the solution volume (L) and m is the mass of apatite powder (g).

**Figure 3:**
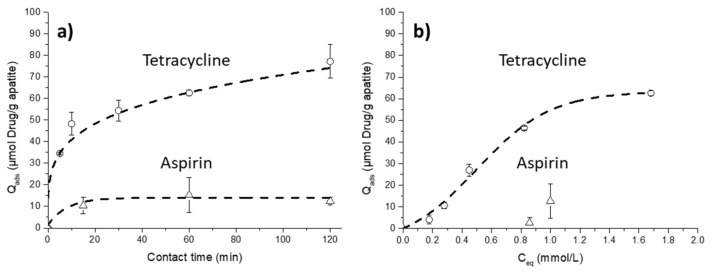
Drug adsorption on biomimetic apatite powder: (**a**) adsorption kinetics at pH = 7.4, room temperature and (**b**) adsorption isotherms at pH = 7.4, room temperature for 1 h; two drug adsorptions are performed with tetracycline (o) and aspirin (Δ) using experimental protocol 1.

Tetracycline (TC) and aspirin (AA) adsorption kinetics measured on the reference biomimetic apatite are reported in Figure 3a. TC adsorption reached quasi-equilibrium in 60 min, while AA adsorption achieved equilibrium in 40 min. The maximum adsorption capacity (Q_m_) was 63 ± 2 µmol/g for TC, which is comparable to values reported in studies performed on apatites [11,59]. The maximum adsorption capacity (Q_m_) for AA was significantly lower, at 11 ± 4 µmol/g. To our knowledge, no study on AA adsorption on biomimetic apatite has been reported previously in the literature. Our findings point to a rather low interaction between AA and apatite surface, even in comparison to TC-like molecules.

For a deeper investigation of the kinetics of drug adsorption on the surface of biomimetic apatite, all data points were tentatively fitted with various kinetic models. For TC and AA adsorptions, the kinetic data were rather well-fitted with an Elovich model. Generally, the Elovich equation (empirical model) is associated with the adsorption of molecules on heterogeneous surfaces [60]. This is the case for biomimetic apatite, which exhibits rather heterogeneous surface features. A fit to the Elovich model was previously found for the adsorption of DNA on biomimetic apatite, for example [61]. The important difference in adsorption between the two drugs can be attributed to their molecular volume, polarity and lipophilicity, as well as the exposed external chemical end-groups (see Table 6). TC has a higher molecular volume than AA and is more hydrophilic and more polar than AA. These latter characteristics may play a role on the molecular diffusion and adsorption rate and capacity and could explain why TC exhibits a higher affinity with biomimetic apatite. TC adsorption on biomimetic apatite has been previously noted [11]. Although this molecule does not exhibit chemical end-groups known to have a high affinity for the surface of apatite, the TC molecule remains amphoteric and polar and involves several ionizable groups that may undergo protonation or deprotonation reactions [11].

The adsorption isotherms report the evolution of the amount of drugs adsorbed, Q_ads_ (µmol/g), on biomimetic apatite as a function of its remaining equilibrated concentration in solution, C_eq_ (mmol/L) (Figure 3b). As observed on the curves, no adsorption isotherm could be obtained for AA experiments on apatite, at least in our working conditions. Furthermore, no AA adsorption was noticed below 0.8 mmol/L (150 ppm), contrary to TC, where adsorption always occurred. AA adsorption on apatite thus did not prove to be efficient in this study. In contrast, TC adsorption led to a well-established isotherm curve. It should be noted that the adsorption isotherm of TC (Figure 3b) has a particular evolution at low concentrations, with the occurrence of an inflection point. This effect may occur particularly towards the low C_eq_ domain in the case of a “Sips” isotherm with an exponent larger than unity (case of positive cooperativity between adsorbing species [11,12]). The Sips isotherm, also named the Langmuir−Freundlich isotherm (Equation (2)), has been proposed and used to model, for example, TC adsorption on biomimetic apatite [11]:

(2) $$Q_{ads}=Q_{m}\times \frac{K_{s}\times C_{eq}^{m}}{1+K_{s}\times C_{eq}^{m}}$$

The mathematical treatment of our data shows that such an equation can describe the evolution of Qads versus Ceq in the case of TC adsorption on biomimetic apatite, with a correlation coefficient of r^2^ = 0.9985 and parameters m = 2.29 ± 0.19, Ks = 0.0034 ± 0.0033 (for C expressed in mg/L) and Qm = 29.81 ± 1.00 mg/g. The good mathematical fit to the Sips model is indicative of a heterogeneous surface, which agrees well with the kinetics study. In addition, some degree of cooperativity between adjacent adsorbed molecules is evidenced by the Sips parameter “m” being noticeably greater than unity. Plotting the adsorption isotherm is also particularly helpful to predetermine the extent of drug loading that will be reached on such biomimetic apatites, depending on the equilibration concentration set in solution, thus making it possible to tailor in a controlled way the loading dose, depending on the intended clinical application and patients’ needs.

#### 2.4.2. Adsorption Kinetics and Isotherms on ACC Material

**Figure 4:**
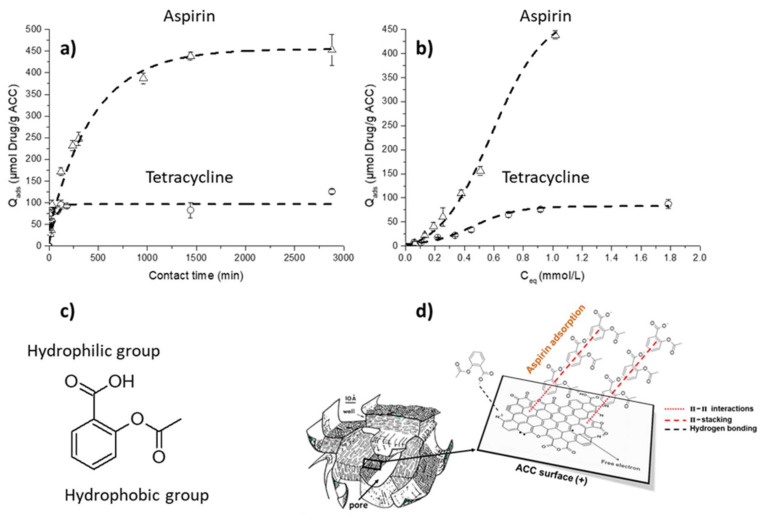
Drug adsorption on activated carbon fiber cloth using experimental protocol 2: (**a**) adsorption kinetics at pH = 7.4, room temperature and (**b**) adsorption isotherms at pH = 7.4, room temperature for 24 h; two drug adsorptions are performed with tetracycline (o) and aspirin (Δ). (**c**) Chemical formula of aspirin, highlighting hydrophobic groups and (**d**) our proposed adsorption mechanism of aspirin at the surface of a porous carbon; model of carbon microtexture proposed by Villey et al. [64].

Similar experiments were performed to study TC and AA adsorption kinetics on ACC. Data are reported in Figure 4a. Tetracycline adsorption reached quasi-equilibrium at the end of 60 min, while aspirin adsorption instead achieved equilibrium at the end of 1440 min (24 h). The maximum adsorption capacity (Q_m_) reached 84 ± 10 µmol/g for TC and 439 ± 9 µmol/g for AA. It should be noted that, for TC adsorption, the drug quantity adsorbed after 24–48 h was <25% compared to the initial quantity placed in solution at t = 0 min, while for AA the quantity adsorbed was >90% of the initial AA amount introduced in solution. These data also show the difference of affinity of the carbon fiber cloth material between AA and TC. All the curves could be adequately fitted with a pseudo first order kinetic model, which was already used for the adsorption of organic molecules [62] and in particular for TC adsorption onto activated carbon cloth [63]. Such a good fit to a pseudo first order kinetic model suggests a relatively simple adsorption process that occurs essentially through diffusion. Previous studies of organic molecule adsorption on ACC material showed the importance of the molecule size and the relationship between the surface charge and the hydrophobicity/hydrophilicity [39,62]. On this material, the organic molecules are mainly adsorbed into the micropores, and in the case of a competition between large and small molecules, the smaller ones are more efficiently adsorbed than the larger ones since they can diffuse more quickly into the ACC microporous network. In addition, hydrophobic molecules may be trapped by the ACC surface via ᴫ-ᴫ dispersive interactions between their aromatic rings and the carbon surface, thus contributing to additional sorption affinity. The small size of the aspirin molecule combined with its hydrophobic characteristics (Figure 4c), along with an adsorption mechanism essentially based on diffusion, could explain the stronger adsorption observed with AA when compared with TC (by a factor of four). Moreover, at pH = 7.4, these molecules are more negatively charged (pK_a_ < pH), while the ACC surface exhibits a positive surface charge (pH solution < pH_PZC_); these experimental conditions may favor drug adsorption via additional hydrogen bonding.

The corresponding isotherms, depicting the evolution of the drug adsorbed amount Q_ads_ (µmol/g) on ACC as a function of its equilibrated concentration in solution C_eq_ (mmol/L), are plotted in Figure 4b. It should again be noted that adsorption isotherms of TC and AA on ACC show a particular behavior at low concentrations, with the occurrence of an inflection point. These adsorption isotherms can also be mathematically fitted with a Sips equation, with a correlation coefficient of r^2^ > 0.998 and m > 1. The hypothesis to explain the adsorption mechanism of these drugs onto ACC may be decomposed into two steps: in a first stage, drug adsorption is likely to occur thanks to π-π dispersive interactions between adsorbed molecules and the ACC surface (with more important adsorption for small molecules like AA compared to TC); then, a positive cooperative synergy between adsorbed molecules may take place thanks to π-stacking (Figure 4d).

These results indicate that, as previously noted for TC adsorption on biomimetic apatite, the sorption of AA and TC on ACC materials may be quantitative and controlled, thus allowing one to predetermine the loading dose as a function of the envisioned medical use.

#### 2.4.3. Adsorption Kinetics on ACC/CDA Material

**Figure 5:**
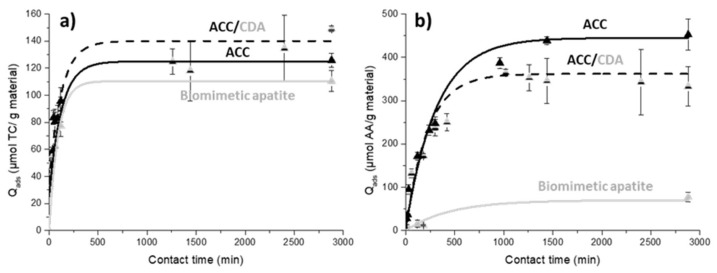
Adsorption kinetics at pH = 7.4 and room temperature on biomimetic apatite (grey line), ACC (black line) and ACC/CDA material (black dash line) for (**a**) tetracycline and (**b**) aspirin.

In order to finalize this adsorption study, the sorption behavior versus TC and AA was evaluated on the final ACC/CDA composite material. The obtained adsorption kinetics of TC and AA were compared with the kinetic curves determined above, separately for biomimetic apatite and ACC (Figure 5).

In the case of tetracycline (Figure 5a), the three materials were found to exhibit rather similar adsorption kinetic evolutions, reaching an adsorbed equilibrated amount between 100 and 140 µmol/g of TC. As expected, the composite ACC/CDA material showed a slightly higher drug adsorption compared to separate ACC and apatite materials, which may be explained by the cumulative tetracycline adsorptions of the apatite coating and the ACC material in the ACC/CDA composite.

For the aspirin adsorption (Figure 5b), the kinetics are rather close for the ACC/CDA and the ACC materials, especially up to 1000 min. Beyond this point, the AA adsorption on ACC/CDA becomes slightly lower than on ACC alone. These results are coherent, taking into account our observation of very low aspirin adsorption on biomimetic apatite in our experimental conditions (see on Figure 3a). Since aspirin was found to have a high sorption affinity with the ACC surface, high Q_ads_ amounts can be ultimately reached in this case. Therefore, AA adsorption on the ACC/CDA composite material becomes progressively limited by the closure of some porosities by the CDA coating (which itself has only a poor degree of interaction with AA).

Overall, these results indicate that co-loading the ACC/CDA composite material with two complementary drugs may be quantitatively achieved in a controlled way.

#### 2.4.4. Double Adsorption on ACC/CDA Material

These findings pave the way for the elaboration of innovative drug release biomaterials for bone applications, capable of associating, in a controlled way, two drugs with complementary properties, by way of modulated sorption features on the two subparts of the ACC/CDA composite.

In order to demonstrate this proof of concept, a “double” adsorption methodology was set up. The drug-loading experiments were performed by following a sequential adsorption approach, using a different adsorption protocol than above (experimental protocol 3, see Figure 10) so as to take into account the possible competitive adsorption between the two drugs. First, aspirin adsorption was performed, which was previously demonstrated to interact almost exclusively with the ACC material. Then, contact with an AA+TC mixture allowed secondary adsorption of TC (while preventing AA undesirable desorption). The final adsorbed amounts (Q_ads_) after completing the entire double adsorption protocol in Figure 10 are reported in Table 4. The same routine was applied to the ACC/CDA composite and to each component separately for comparative purposes. At the end of this adsorption experiment, the aspirin amount adsorbed on ACC/CDA material (Q_ads_ = 2.10 ± 0.10 mmol/g) was found to be close to the amount adsorbed on the pure ACC material (Q_ads_ = 2.07 ± 0.06 mmol/g). Tetracycline was also, as expected, adsorbed on the ACC/CDA composite. The final adsorbed TC amount corresponded to the amount of TC adsorbed on the apatite component (0.07 ± 0.01 vs. 0.06 ± 0.01 mmol/g), suggesting that TC was, in these conditions, essentially adsorbed on the CDA coating deposited on the ACC surface. Consequently, this allowed obtaining a dually-loaded ACC-AA/CDA-TC biomaterial.

#### 2.4.5. Desorption/Release Study

**Figure 6:**
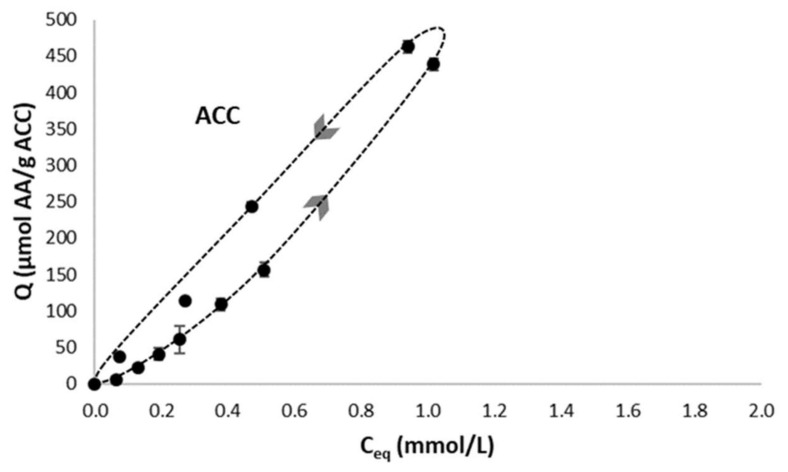
Aspirin adsorption-desorption isotherms on ACC material.

Aspirin showed a high retention by the ACC cloth but very limited sorption on apatite in our experimental conditions. Therefore, upon immersion (e.g., after implantation), its release is likely limited by diffusion through the porous network up to the surrounding medium. A rather straightforward desorption scheme is thus to be expected in this case, as was observed (Figure 6).

**Figure 7:**
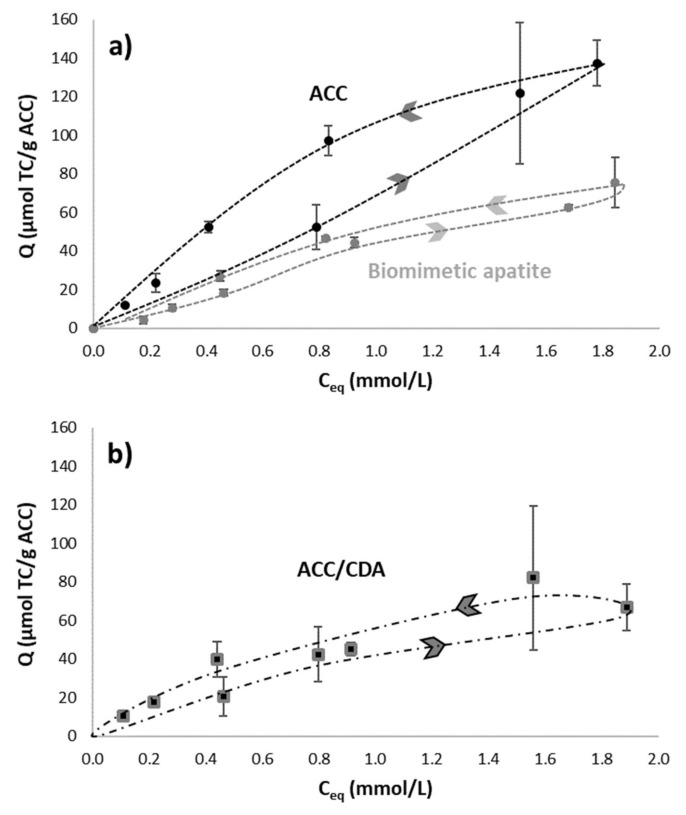
(**a**) Tetracycline adsorption-desorption isotherms on ACC materials (black dash line) and on biomimetic apatite powder (grey dash line). (**b**) Tetracycline adsorption-desorption isotherms on ACC/CDA material.

On the other hand, the case of TC is more complex, as it has an affinity for both apatite and ACC. Therefore, it was interesting to study how the association of this drug to each of the two components of the ACC/CDA composite material could modulate the desorption features. To do this, adsorption-desorption tests were performed on the biomimetic apatite powder (grey dash line) and on ACC material (black dash line), as reported on Figure 7a. The adsorption-desorption cycles clearly depict two distinct modes of desorption.

Concerning the biomimetic apatite powder, the TC desorption profile followed a curve close to that of the adsorption process: an almost reversible phenomenon was observed with a very narrow hysteresis. This result may indeed be expected as a “simple” desorption scenario (molecular detachment from the surface without additional modifications of the apatite substrate), as was reported for the desorption of enzymes from biomimetic apatite [24]. This is not surprising, taking into account the absence of end-groups known to have a high affinity for apatite surfaces (in contrast to bisphosphonates).

On the contrary, for the ACC material, TC desorption followed a different route, leading to a well-defined hysteresis, with the desorption curve well above the adsorption isotherm. These findings illustrate a situation where desorption is partially hindered upon dilution, which can be explained here by the porous network of the ACC cloth providing diffusional limitations to desorption.

The difference in desorption behavior of the two components of the ACC/CDA composite materials may be seen as an appealing characteristic of such biomaterials, by exhibiting two distinct pathways for the release of a wide-spectrum antibacterial agent such as tetracycline. While apatite is expected to release TC directly upon contact with the surrounding (body) fluids, thus generating a first antibacterial effect, a second release may be expected for the TC molecules entrapped in the porosity of the ACC material, in view of a delayed effect after implantation. The actual drug release from the ACC/CDA composite material is shown in Figure 7b, evidencing, as expected, releasing features close to those of each component taken separately.

**Figure 8:**
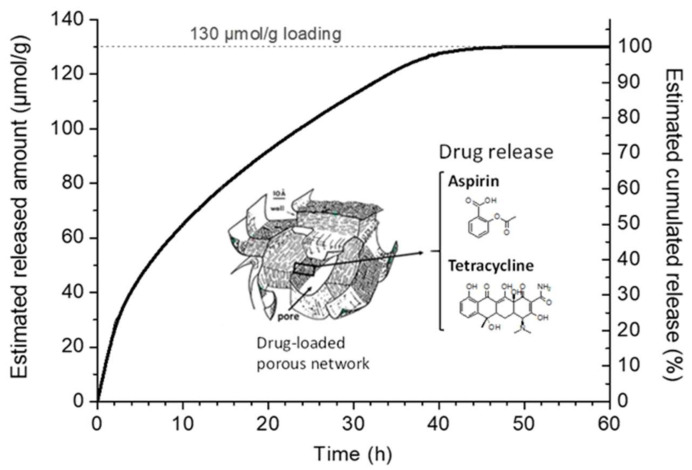
Tetracycline and aspirin estimated release from ACC substrate.

From the above, it may be concluded that (i) neither of the two drugs considered here exhibits a high affinity for the substrate and (ii) the porous network is bound to control the diffusion of chemical species. Therefore, the drug(s) release properties are likely governed essentially by diffusivity across the porosity. A previous study on TC adsorption/release from a biomimetic apatite scaffold (with intrinsic porosity) showed that the release could well be described, at least in a first good approximation, on the basis of Fickian diffusion in √t corresponding to the Higuchi model (a particular case of the Korsmeyer−Peppas model) [11,65]. Based on the above considerations, it appears reasonable to also expect the release kinetics in the present case to follow a Higuchi model. The estimated curve for the Higuchi-modeled cumulated release is proposed in Figure 8. This is in line with experimental titrations on AA- or TC-loaded scaffolds, confirming, in particular, a total release of the 130 µmol/g dose (see Table 5) at the time point of 48 h. Therefore, in the conditions presented in this paper, the system presented may be used in view of a rather fast release, starting at the time of implantation and during the first days after surgery.

### 2.5. In Vitro Biocompatibility Assays on Human Osteoblasts

In vitro biological experiments were performed using human osteoblast cells (HOST) on the three composite materials, loaded or not by each of the AA/TC drugs: (1) ACC/CDA material (control group), (2) ACC/CDA-TC and (3) ACC/CDA-AA (Table 5). The drug amounts selected for this experiment were approximately 130 µmol/g (corresponding to 500 µg of TC and 200 µg of AA, per specimen of material tested in vitro). After 2 days of culture, cell viability was analyzed from fluorescence microscopy images (see details in Section 2.3.). The number of live cells and dead cells, as well as r, the ratio between both, is reported in Table 5.

A difference in osteoblast number is clearly observed between the ACC/CDA control material and the drug-loaded materials (ACC/CDA-TC and ACC/CDA-AA). The presence of tetracycline does not modify the live cell number on the material surface, but it strongly decreases the dead cell number. On the contrary, the use of aspirin strongly increases the number of live cells and does not modify the dead cell number. For both, the ratio “r” strongly increases in the presence of the drugs. These results emphasize that the loading of ACC/CDA material with therapeutic molecules such as tetracycline or aspirin leads to beneficial effects on the viability of the primary human bone cells. Coupling these therapeutic molecules with ACC/CDA material thus appears highly promising for an application in bone regeneration.

### 2.6. In Vivo Effect of ACC/CDA-AA Material on Cortical Bone Regeneration

The in vivo experiments performed in this work aimed at testing the capacity of ACC/CDA material to be used as a bone repair patches exhibiting drug delivery properties. A special focus was on the association with aspirin and its in situ release, in view of a proof of concept showing local drug release capacity of such ACC/CDA composites (i.e., ACC/CDA-AA material).

**Figure 9:**
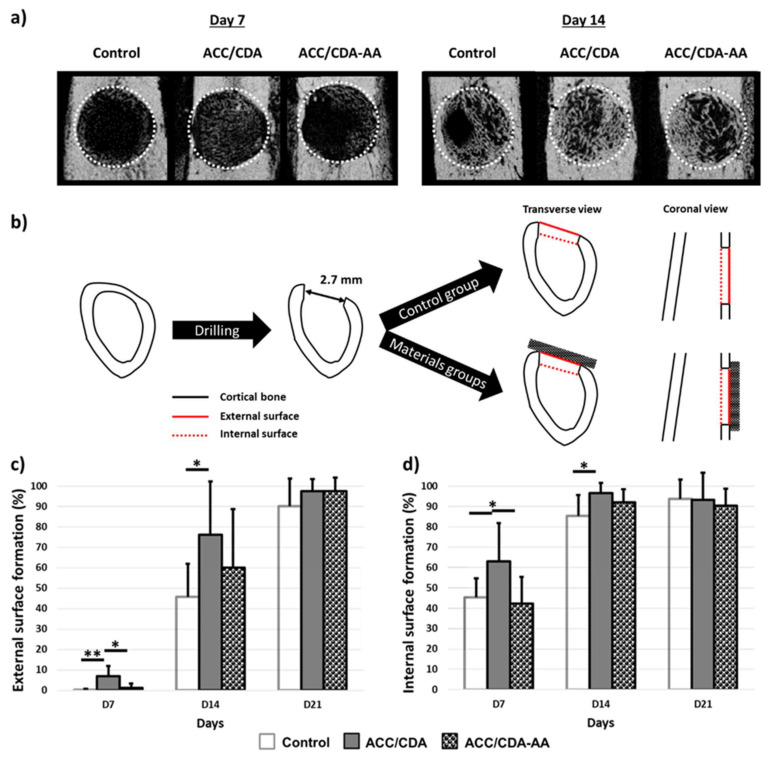
Further analysis of a cortical reconstruction at days 7, 14 and 21 post-surgery. (**a**) Higher magnification of µ-CT scan pictures of the representative samples for each group at days 7 and 14. Bone defect is delimited by a white dotted line. (**b**) Schemes describing the evaluation of the bone formation at the external and internal surfaces of the cortical bone. (**c**,**d**) Quantification of new cortical bone formation (**c**) at the external surface and (**d**) at the internal surface. Mean ± standard deviation, * *p* < 0.05, ** *p* < 0.01.

Cortical bone reconstruction was analyzed by µCT (Figure 9a), in particular focusing on the external (Figure 9c) and internal surfaces (Figure 9d), as schematized in Figure 9b. Bone regrowth quantification showed that the presence of the ACC/CDA material significantly accelerated the external cortical surface reconstruction at days 7 and 14, as compared with the control group (6.8% vs. 0.2% and 76.2% vs. 45.83%). The aspirin-loaded ACC/CDA-AA material was also found to accelerate the external surface reconstruction at day 7 and at day 14, as compared to the control group (1.2% vs. 0.2% and 60.0% vs. 45.8%), although with slower kinetics than the AA-free composite (1.2% vs. 6.8% at day 7 and 60.0% vs. 76.2% at day 14). At day 21, however, no statistical difference could be noticed between the three groups (Figure 9b). In the same way, measurements of the internal surface of the cortex indicated that, at day 7 and day 14, the ACC/CDA material significantly accelerated bone formation as compared to the control group (63.1% vs. 45.2% and 96.6% vs. 85.44%). Here again, the presence of AA in the ACC/CDA material led to a slowdown of the reconstruction as compared to the unloaded ACC/CDA material (42.2% vs. 63.1% at day 7 and 92.1% vs. 96.6% at day 14). However, at day 21, no significant difference was observed between the three materials (Figure 9c), showing notable bone regrowth.

First, quantification of bone reconstruction from external and internal surfaces showed the strong effect played by the apposition of the ACC/CDA material for bone reconstruction. It was well-marked from day 7 and at day 14. This shows that the ACC/CDA material significantly accelerated the bone regeneration. Second, for the ACC/CDA material in which aspirin was adsorbed, the results showed a direct effect of the presence of aspirin, since both the external and internal surfaces were impacted despite a depth of about 500 µm (i.e., the depth of the cortical bone defect). The evidence of aspirin’s effect in spite of this large distance clearly reveals that aspirin was locally released at the vicinity of the implanted materials and did not remain blocked in the porosity. Although quantification of AA release in these in vivo experiments could not be realized to titrate the amount of AA released, these findings emphasize that this ACC/CDA-AA system was efficient for the in situ release of aspirin, used here as a model drug. This is an important result, and it encourages us to perform in future works in vivo biological tests with other bioactive molecules so as to widen therapeutic opportunities. Concerning the bone reconstruction itself, a slowing down of the bone repair process was nonetheless noted compared to the raw undoped ACC/CDA material, though bone reconstruction was clearly observed.

Beyond being a model drug in this work, the choice of aspirin is not trivial and may by itself appear promising for further explorations in association with such ACC-based bone patches. Indeed, recent studies have suggested its potential to promote bone regeneration [51,52,53,54,55,56], which might be linked to its anti-inflammatory activity, among other properties. In these papers, various biomaterials containing aspirin were implanted on calvaria, mandibular or periodontal defects but never on long bone defects. In our present work, in vivo experiments were performed on the diaphysis of a rat femur (a long bone), which is well known to have an important vascularization. Some papers pointed out the importance of blood clot occurrence during the bone regeneration processes [66,67]. Our understanding of our results is that aspirin probably leads to a reduction in platelet aggregation, giving rise to a longer formation of blood clots, which generates a slowdown in the overall bone regeneration. Nonetheless, our work shows for the first time the possibility to use an activated carbon fiber cloth coated with CDA as a bone patch able to release, in situ, a bioactive molecule or a combination of molecules—as exemplified by the AA/TC duet directly around the bone defect—quickly and efficiently, so as to provide additional therapeutic effects. This work paves the way for doping of such ACC/biomimetic apatite composite materials with a wealth of other therapeutic agents in view of conveying tailored medical functionalities for bone tissue repair.

## 3. Materials and Methods

### 3.1. Materials

#### 3.1.1. Apatites

The “reference” nanocrystalline apatite, namely the biomimetic apatite powder used in this study, was synthesized following the procedure described by Vandecandelaere et al. [57], which consists of a precipitation method performed at room temperature and close to physiological pH (7.4), with the latter being ensured by an excess of phosphate ions in aqueous medium. Two starting solutions were used: solution A consisted of calcium nitrate tetrahydrate (Merck Emsure grade, purity 99.0%) as the calcium source (calcium concentration of 0.3 M), and solution B consisted of diammonium hydrogenphosphate (VWR Normapur grade, purity 99.0%) as the phosphate source (phosphate concentration 0.6 M). After rapidly pouring solution A into solution B and mixing, the precipitate was left to mature for 1 month prior to filtration on a Büchner funnel, washing with deionized water, and freeze-drying. This led to a powder sample of nanocrystalline apatite for use in the drug adsorption study.

The biomimetic apatite coating on the ACC substrate was performed at room temperature using a sono-electrodeposition process [45,46,47]. The electrolyte consisted of a mixture of calcium nitrate tetrahydrate Ca(NO_3_)_2_, 4H_2_O (Sigma-Aldrich, BioXtra, purity 99.0%), and ammonium dihydrogenphosphate NH_4_H_2_PO_4_ (Alfa Aesar, purity 98.0%), maintaining a Ca/P ratio of 1.67 with [Ca^2+^] = 5 mmol/L. The initial pH was kept at 4.8. An electrochemical cell was home-made to use ACC as the working electrode, a platinum basket surrounding the ACC was used as the counter electrode and Hg/Hg_2_SO_4_ (Radiometer analytical, France) was used as the reference electrode. The cathodic polarization of the carbon electrode was performed using a potentiostat/galvanostat (Biologic Science Instruments VMP-2, France) with a constant potential of −1 V/Hg/Hg_2_SO_4_, which is the theoretical barrier potential of water reduction at initial pH = 4.8. Sonication (35 kHz, 30%) was applied during polarization (ultrasonic bath, Transsonic Ti-H-10, Fisher Scientific France). The experimental conditions were optimized in a previous work [46]. This led to the formation of a biomimetic apatite consisting of a carbonated calcium-deficient hydroxyapatite (CDA) phase deposited on a ACC substrate, as we reported previously [47].

Ca/P atomic ratios were determined by Energy Dispersive X-ray Spectroscopy (EDS) analysis performed in Transmission Electron Microscopy (TEM, TEM-PHILIPS CM20) operating at 200 kV. The atomic ratios were calculated from the intensities of the Ca peak (Kα Ca = 3.691 keV) and P peak (Kα P = 2.015 keV) present in the TEM-EDS spectra.

The crystallographic structure of the CaP coatings was analyzed by X-ray diffraction (XRD) using an INEL diffractometer (CSP120) working at 40 kV and 30 mA (Cu Kα = 1.5418978 Å). Data were collected in the range of 2θ = 10–80°, with a curve detector (angular instrumental resolution of 0.05°), in transmission mode, using a Si(111) monochromator. To determine the lattice parameters of CaP crystalline phases, refinements were performed by the Lebail method using Jana2006 software.

#### 3.1.2. Activated Carbon Fiber Cloth (ACC) Substrates 

The three ACC substrates selected for this study were referred to as ACC 1, ACC 2 and ACC 3. They were respectively provided by Dacarb^®^, Zorflex^®^ and Mast Carbon^®^. Prior to their use, the ACC substrates were washed with boiling distilled water, using a Soxhlet^®^ extractor to remove any traces of contaminants coming from the activation and carbonization steps in their manufacturing. After 12 h washing, they were dried at 70 °C under vacuum.

The porosity of ACC substrates was characterized by N_2_ adsorption at 77 K, using an Autosorb-1 (Quantachrome). The total pore volume was estimated from the amount adsorbed at P/P° = 0.95. The micropore (pore size < 2 nm) and mesopore (2 nm < pore size < 50 nm) volumes were determined by applying the density functional theory (DFT) method or Dubinin-Radushkevich (DR) theory to the N_2_ isotherms, assuming a slit pore shape. The pH value corresponding to an ACC net charge of zero, namely pH_PZC_, was measured at constant ionic strength in NaNO_3_ (0.01 mol/L). The amount of acidic functional groups grafted on the ACC surface was measured by potentiometric titration with NaOH (0.1 mol/L), using a very low incremental volume (0.01 mL) in a wide range of pH (3–11).

#### 3.1.3. Drugs

Two drugs were used in this work. The commercial antibiotic agent used in this work was tetracycline (TC) in the form of the rather soluble tetracycline phosphate salt (Sigma, CAS No. 1336-20-5): 100 mg of compound containing 82 mg of TC. The commercial NSAID used was acetylsalicylic acid (AA, Aspirin; Sigma, CAS No. 50-78-2). All drug concentrations given in this paper refer to the pure drug and correspond to milligrams of drug per liter (= ppm). The main characteristics of tetracycline and aspirin are given in Table 6.

### 3.2. Drug Adsorption and Release Experiments

#### 3.2.1. Drug Adsorption on Biomimetic Apatite Powder

For each drug, the adsorption study was performed in deionized water containing 10^−2^ M KCl, for providing a rather constant ionic force during the adsorption, and at physiological pH = 7.4. Experiments were run at room temperature (~22 °C), corresponding to conceivable conditions for the preparation of drug loaded systems in a potential pharmaceutical company and also for facilitating the preparation of samples, including for the in vivo experiments. The drug adsorption study on biomimetic apatite powder was carried out in two steps, as described below (experimental protocol 1).

At first, a kinetic survey was carried out in order to determine the minimal amount of time needed to reach the thermodynamic adsorption equilibrium. To this end, several time points were examined, between 5 and 120 min, using in each case 20 mg of apatite suspended in 5 mL of drug solution (820 ppm for TC and 180 ppm for AA). After the selected duration, the sample was immediately isolated from the supernatant by centrifugation (2 min at 5000 rpm), and the supernatant was titrated by UV spectrophotometry (details given below).

Then, the drug adsorption isotherm on the biomimetic apatite powder was established with the same pH, solid/liquid ratio and temperature (22 °C) as for the kinetic study, but varying the initial drug concentration (for a contact time of 60 min). As above, each sample was centrifuged, and the drug concentration in the supernatant was titrated by UV spectrophotometry.

In each case, the amount of drug adsorbed on the apatite powder was determined by the difference between the initial drug concentration and the final concentration in the supernatant.

The drug concentration in solution was titrated by UV spectrophotometry (Agilent technologies, Cary 60 UV-Vis), by monitoring the optical density at the drug absorption maximum, in relation to a calibration curve previously established. Tetracycline in solution was titrated by monitoring the optical density at 355 nm (adsorption maximum), in relation to a calibration curve established between 0 and 820 mg/L of TC (correlation coefficient 0.9996). Aspirin in solution was titrated by monitoring the optical density at 277 nm (adsorption maximum), in relation to a calibration curve established between 0 and 180 mg/L of AA (correlation coefficient 0.9998).

#### 3.2.2. Drug Adsorption on ACC and ACC/CDA Composite Materials

The adsorption experiments on ACC-based materials were performed in the same conditions as for apatite powder: in deionized water supplemented by 10^−2^ M KCl, at a pH = 7.4 and at room temperature (~22 °C). Drug adsorption tests on 1 cm patches of ACC and ACC/CDA materials were also carried out in two steps, as for the apatite adsorption study (see Section 2.2.). The contact time with drugs ranged from 5 to 2880 min for the kinetic survey and was set to 1440 min for determining the adsorption isotherm (experimental protocol 2).

For each experiment, the amount of drug adsorbed on the ACC or ACC/CDA materials was determined by the difference between the initial drug concentration and the final concentration in the supernatant. The drug concentration in solution was again titrated by UV spectrophotometry. All experiments were performed in triplicate.

#### 3.2.3. Double Drug Adsorption on ACC/CDA Composite Material

**Figure 10:**
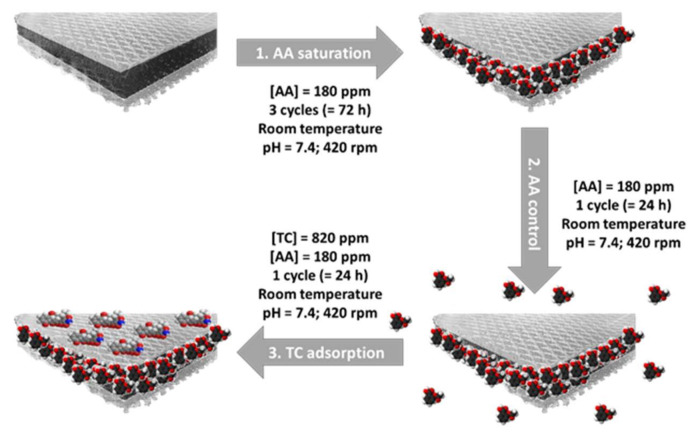
Double adsorption protocol on ACC/CDA composite material.

Experiments were made to associate both AA and TC to ACC/CDA composite materials in view of an innovative dual therapeutic approach. First, the ACC/CDA composite material was put under vacuum in a reactor for 2 h. Then, the reactor was filled with deionized water in order to impregnate well the ACC substrate porous network, for 24 h. Afterward, the ACC/CDA material was saturated with aspirin solution (180 ppm) by way of 3 adsorption cycles of 24 h at pH = 7.4 and at room temperature. Next, a fourth cycle was added to confirm the AA saturation under these conditions. In a third step, a fifth cycle was performed with a solution containing both aspirin (180 ppm) and tetracycline (820 ppm). The presence of AA in this solution was selected to ensure the absence of unwanted AA release upon TC adsorption (verified by subsequent titrations). The protocol of this double adsorption is summarized in Figure 10 (experimental protocol 3). After each step, the corresponding amount of drug was measured by UV spectrophotometry.

#### 3.2.4. Drug Release

Drug release on the previously doped materials was determined as follows. From the most concentrated solution (i.e., solution corresponding to the last data point of the adsorption isotherm), half of this solution was removed and replaced by a neutral buffer solution (pH = 7.4, KCl 10^−2^ M, T_amb_). After the selected contact time (60 min), the drug concentration in the supernatant was titrated by UV spectrophotometry. The experiment was repeated to obtain data points for different concentrations leading to the desorption isotherm. All experiments were performed in triplicate. The shape and the location of the characteristic peaks (AA or TC) were systematically verified in order to confirm the absence of drug degradation (Figure S2).

### 3.3. In Vitro Procedures

Materials were cut in order to obtain patches of 8 mm diameter, which were sterilized by heat drying for 2 h. Cell viability and proliferation assessments were conducted in two different assays: first, on three ACC materials, namely ACC 1, ACC 2, ACC 3 (see Table 2); second, on the three other materials developed in this work: ACC/CDA, ACC/CDA-TC and ACC/CDA-AA materials (see Table 5). ACC/CDA-TC and ACC/CDA-AA drug adsorption was carried out in conditions similar to the adsorption study. The drug solutions were prepared at 820 ppm and 180 ppm for TC and AA, respectively, and sterilized using a 0.2 µm filter. ACC/CDA patches were immerged in 10 mL of these solutions for 1 h just before the biological experiments.

For human primary osteoblast (HOST) tests, material patches of 8 mm diameter were pre-incubated in 48-well culture plates in α-MEM medium supplemented with 10% fetal calf serum, 100 U/mL penicillin, 100 U/mL streptomycin and 2% ultraglutamine (complete medium) and incubated at 37 °C under 5 % CO_2_ for 6 h. Then, human primary osteoblasts were seeded in complete medium either at 10,000 cells/well (first experiment) or 7000 cells/well (second experiment) for 2 days. Cell viability and proliferation were then analyzed. The human primary osteoblasts were purchased from PromoCell (Heidelberg, Germany).

Cell viability was assessed using the fluorescent assay LIVE/DEAD^TM^ Viability/Cytotoxicity Kit (Life Technologies SAS, France). At each endpoint, ACC patches were gently washed with phosphate-buffered saline (PBS) and incubated at room temperature with PBS containing 2 µmol/L calcein-AM and 4 µmol/L ethidium-homodimer-1. Calcein-AM is a nonfluorescent cell-permeant fluorescein derivative, which is converted by cellular esterase activity into cell-impermeant and highly fluorescent calcein. Calcein accumulates inside live cells having intact membranes, which results in a green fluorescent signal. Ethidium-homodimer-1 enters dead cells with damaged membranes and undergoes a 40-fold enhancement of fluorescence upon binding to their DNA, leading to a red fluorescent signal. After 20 min incubation, the ACC patches were put onto a glass slide, covered with a coverslip and observed under a fluorescence microscope (Zeiss Observer Z1 with FITC filters and Texas Red) equipped with FITC and Texas Red filters. The number of live cells was estimated using ImageJ Software.

### 3.4. In Vivo Procedures

#### 3.4.1. Surgical Procedure

To investigate the possibility of using ACC/CDA material as a drug delivery system in a bone context, biomaterials developed in this study were tested in a rat femoral defect model. All the animal experiments received the approval of the local committee and the French Ministry of Higher Education, Research and Innovation on 20 March 2019 (Authorization APAFIS#19215-2019021512224201).

Anesthesia induction was done by inhalation of 5% isoflurane and maintained by intramuscular injection of 30 mg/kg ketamine (Virbac, Carros, France) and 7.6 mg/kg xylasine (Dechra, Montigny-le-Bretonneux, France), completed by a subcutaneous injection of 0.1 mg/kg robinul-V (Vetoquinol, Magny-vernois, France) and by intraperitoneal injection of 0.036 mg/kg buprenorphine (Bubrecare^®^, Axience, Pantin, France).

The operation was performed in strict aseptic conditions. The rats were positioned in lateral decubitus and maintained under a mixture of oxygen (2 L/min) and air (5 L/min). A bilateral cylindrical cavity was created in the distal femoral metaphysis. First, the two limbs were shaved, the skin was disinfected with iodine solution and the animal was sterile draped. After lateral skin incision, the distal part of the femur was exposed. A cylindrical defect 2.7 mm in diameter and 4–5 mm in depth, including the periosteum, was created using a motor-driven drill (Advetis Medical, Montreuil, France) and successive burs of 1.1, 1.5 and 2.7 mm diameter. During drilling, the site was irrigated using sterile saline. Bone reaming debris was removed by saline irrigation. The defect was then covered with an ACC/CDA or ACC/CDA-AA material, except in the control group, where it was left uncovered (spontaneous regeneration). Surrounding structures were then guided back into their natural anatomic positions until muscles and skin were closed by sutures.

#### 3.4.2. AA Adsorption during Surgical Procedure

Just before surgery, the ACC/CDA-AA material was prepared by immersing a 10 mm × 4 mm ACC/CDA patch in 10 mL of aspirin sterilized solution (600 ppm) for 10 min at room temperature. As measured by spectrophotometry, this step led to the adsorption of 250 ± 15 µg (i.e., 165 ± 10 µmol/g) of aspirin in the ACC/CDA material.

### 3.5. Micro-Computed Tomography Analysis

After 7, 14 and 21 days post-surgery, the rats were sacrificed with carbon dioxide, and the femurs were dissected and fixed in 10% buffered formalin. Micro-computed tomography imaging of the whole femur was performed using a high-resolution µCT scanner (Skyscan 1173, Synergie4, Evry, France) at a voltage of 90 kV, current of 88 mA, isotropic pixel size of 12.1 µm, exposure time of 250 ms and a 1 mm thick aluminum filter for beam hardening reduction. Three-dimensional reconstructions of the defect zones were obtained using N-Recon software. For orientation purposes, to define the region of interest and for the 3D image analysis, CTAn software (Bruker) was used. The CTAn software was also used to quantify the ratio of new bone for each region of interest.

### 3.6. Statistical Analyses

Micro-computed tomography data were statistically analyzed using the Student t-test, and differences were considered statistically significant at * *p* < 0.05 or ** *p* < 0.01. For each group *n* = 7 or *n* = 8.

## 4. Conclusions

In this study, the adsorption of tetracycline and aspirin on biomimetic apatite, activated carbon fiber cloth (ACC) and ACC/CDA composite material was performed and analyzed. Adsorption on each component of the biomaterial showed the importance of the adsorbed molecule/adsorbent affinity. Tetracycline, being a polar and hydrophilic molecule, is the preferred adsorbed molecule for biomimetic apatite. Aspirin, being a smaller and hydrophobic molecule, is the preferred adsorbed molecule for ACC. The adsorption and desorption of both drugs were studied, highlighting drug release specificities between CDA and ACC loaded materials and the propensity to apply this approach to actual ACC/CDA composites. To go one step further, an ACC/CDA material was co-loaded with the two drugs, leading to a double adsorption functionality in the form of innovative ACC-AA/CDA-TC biomaterial. In vitro tests evidenced better osteoblast viability and proliferation for the drug-loaded (tetracycline or aspirin) ACC/CDA materials than their drug-free counterpart. In vivo experiments were run on a rat model of large cortical bone defect, highlighting the possibility to use such activated carbon fiber cloth coated with biomimetic nanocrystalline apatite for significantly accelerating bone reconstruction. By taking advantage of the specific porous and surface-reactive characteristics of such materials, it is possible to envision the development of bone patches with drug delivery properties in view of the in situ release of therapeutic drug(s) to improve bone healing, as we have demonstrated in this proof of concept.

## Figures and Tables

**Table 1:** Characteristics of the “reference” apatite and carbonated CDA deposited on ACC substrate.

	$\frac{Ca}{P}\;\mathrm{Atomic}\;\mathrm{Ratio}\;\;(\mathrm{SEM}-\mathrm{EDX})$	CO_3_ %wt.	Morphology (SEM)	Structure (XRD)	Lattice Parameters	
					a (Å)	b (Å)
“Reference” nanocrystalline apatite	1.46 ± 0.02	0	Plate-like	Hexagonal, (P6_3_/m)	9.437 ± 0.004	6.871 ± 0.002
Carbonated CDA	1.4 ± 0.1	2–6	Plate-like	Hexagonal, (P6_3_/m)	9.366	6.820

**Table 2:** Morphological, physico-chemical and textural characteristics of the three ACC materials.

	Precursor	Sizing	Architecture	Fiber Diameter (µm)	S_BET_ (m^2^/g)	V_TOTAL_ (cm^3^/g)	Acidic Functional Groups (mmol/g)	pH_pzc_
ACC 1	Phenolic resin	–	Woven	10	1693	0.68	0.48	9.4
ACC 2	Viscose	ZnAl_2_O_4_	Knitted	8–12	898	0.51	2.93	7.8
ACC 3	Viscose	ZnAl_2_O_4_	Knitted	12–15	1400	1.27	1.31	5.8

S_BET_ = Specific surface area calculated from the N_2_ isotherm using the method; V_TOTAL_ = total pore volume estimated from the N_2_ isotherm; pH_pzc_ = pH value corresponding to an ACC net charge of zero.

**Table 3:** Textural characteristics of ACC (FM50K) and ACC/CDA materials analyzed by N_2_ adsorption at 77 K.

	S_BET_ (m^2^/g)	V_micro_^1^ N_2_, DFT (cm^3^/g)	V_meso_^1^ N_2_, DFT (cm^3^/g)	V_micro_^2^ N_2_, DR (cm^3^/g)
ACC	898	0.41	0.07	0.41
ACC/CDA	980	0.45	0.10	0.46

^1^ The microporous and mesoporous volumes are determined by applying the density functional theory method (DFT) to the N_2_ adsorption isotherms. ^2^ The microporous volume is determined by applying the Dubinin-Radushkevich theory (DR) to the N_2_ adsorption isotherms.

**Table 6:** Chemical formula and main characteristics of TC and AA.

	Chemical Formula	M (g/mol)	Volume (Å^3^)	pKa (25 °C)	Log P	Log D (pH = 7.4)	Polar Surface Area (Å^2^)
Tetracycline (TC)	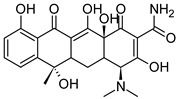	444.43	449	3.3	−1.47 ± 0.81	0.48	182
Aspirin (AA)	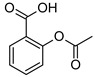	180.16	232	3.5	1.19 ± 0.23	2.93	64

**Table 4:** Comparison of TC and AA adsorbed amounts on ACC, biomimetic apatite powder and the composite ACC/CDA material (following protocol 3 as depicted in Figure 10).

	AA Adsorption	TC Adsorption
	ACC	Apatite
Q_ads_ (mmol/g)	2.07 ± 0.06	0.06 ± 0.01
	ACC/CDA	
Q_ads_ (mmol/g)	2.10 ± 0.10	0.07 ± 0.01

**Table 5:** Live or dead osteoblast cell number calculated using fluorescence microscopy images after coloration by live/dead kit. Quantification of cell number by mm^2^ on the materials after 2 days of culture was performed using the ImageJ software. Mean ± standard deviation.

	Drug Loading		Live/Dead Assay		
	Q_ads_ (µmol/g)	Q_ads_ (µg)	Live Cells/mm^2^	Dead Cells/mm^2^	“r” (L/D)
ACC/CDA	-	-	18 ± 4	14 ± 2	1.30
ACC/CDA-TC	133 ± 36	500 ± 130	18 ± 9	6 ± 2	3.00
ACC/CDA-AA	134 ± 23	200 ± 30	51 ± 13	14 ± 2	3.65

## Data Availability

All the data used and/or analyzed for the current study is contained in the article. All other datasets are available from the corresponding author upon reasonable request.

## Supplementary Materials

The following are available online at https://www.mdpi.com/article/10.3390/ijms222212247/s1.
