# Supplementary material for: Activated Carbon Fiber Cloth/Biomimetic Apatite: A Dual Drug Delivery System
Source: Int J Mol Sci. 2021 Nov 12;22(22):12247. doi: 10.3390/ijms222212247 (PMC8624510; doi:10.3390/ijms222212247)
Supplement: Supplementary file 1 [file ijms-22-12247-s001.zip › ijms-1430594-supplementary.pdf]

Supplementary Information

SUPPLEMENTARY INFORMATION INDEX

- Supplementary Figure 1S
- Supplementary Figure 2S

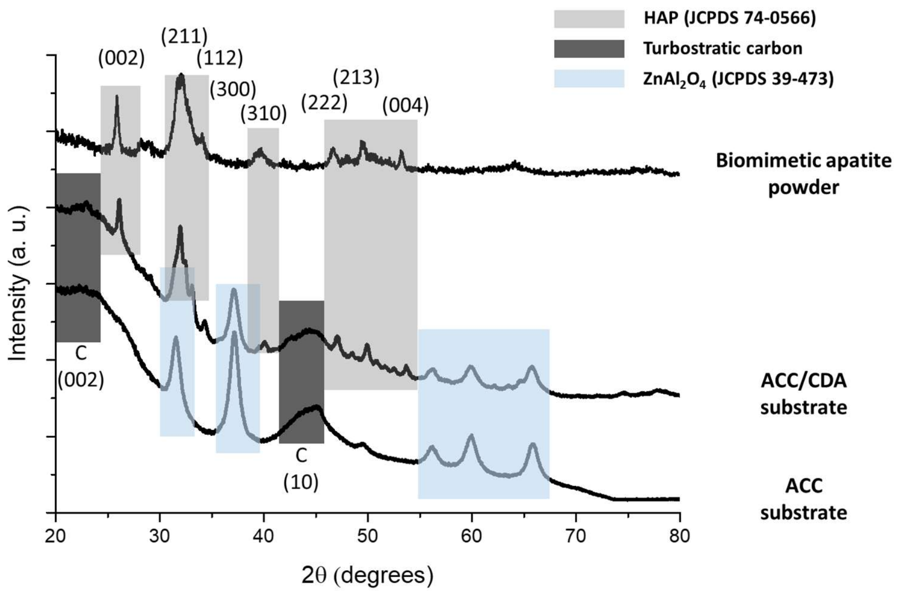

Figure 1S. XRD diffractograms of ACC, ACC/CDA and biomimetic apatite materials.

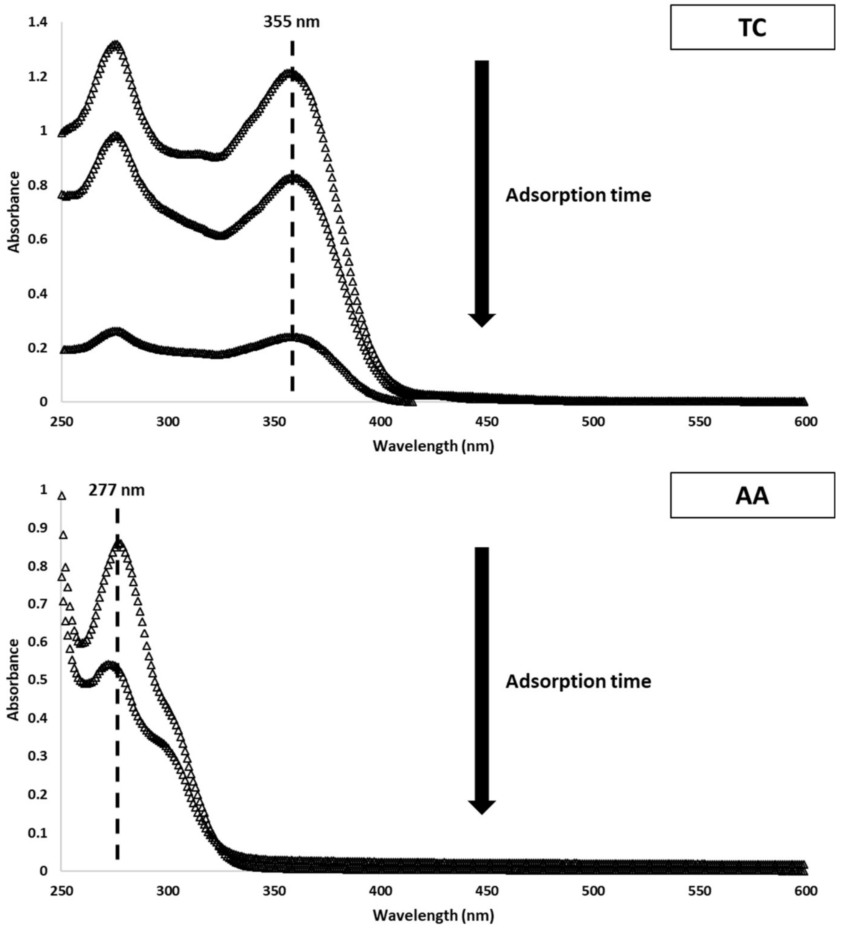

Figure 2S. UV absorbance spectra of tetracycline (TC) and aspirin (AA) at different adsorption time points.
